# Optimized Preparation of Nanosized Hollow SSZ-13 Molecular Sieves with Ultrasonic Assistance

**DOI:** 10.3390/nano10112298

**Published:** 2020-11-20

**Authors:** Liang Zhou, Runlin Han, Yuxuan Tao, Jinqu Wang, Yiwei Luo

**Affiliations:** 1 School of Chemical Engineering, Dalian University of Technology, Panjin 124221, China; zhouliang@dlut.edu.cn (L.Z.); tyxlogo@163.com (Y.T.); wangjq@dlut.edu.cn (J.W.); lyw890816@hotmail.com (Y.L.); 2 School of Chemistry and Chemical Engineering, Jinggangshan University, Ji’an 343009, China

**Keywords:** SSZ-13, CHA zeolite, hollow molecular sieves, CO_2_ adsorption

## Abstract

Because of its unique eight-membered ring pore structure and the arrangement of cations in its structure, the SSZ-13 molecular sieve has a higher affinity for CO_2_ than other gases, meaning it has attracted more attention than other porous materials for CO_2_ adsorption. However, the expensive template and long preparation time limits the industrial production of SSZ-13. In this work, a hollow structure was successfully introduced into the nanosized SSZ-13 molecular sieve with ultrasonic treatment. The effects of the amount of seed added and the ultrasonic time on the structure were investigated. When the amount of seed added was 0.5 wt.% and the ultrasonic time was 60 min, the sample showed a hollow cubic crystal with a diameter of about 50 nm. The specific surface area reached 791.50 m^2^/g, and the mesoporous ratio was 66.3%. The samples were tested for CO_2_ adsorption performance at 298 K. It was found that the hollow sample prepared in this work has higher CO_2_ adsorption capacity compared with the SSZ-13 zeolite prepared with conventional methods. When the adsorption pressure was 0.27 bar, the adsorption amount reached 2.53 mmol/g. The hollow SSZ-13 molecular sieve reached a CO_2_ adsorption capacity of 4.24 mmol/g at 1 bar.

## 1. Introduction

The emission of greenhouse gases due to burning fossil fuels causes serious global warming, which is one of the most pressing issues in the world. To address this issue, the development of facile and cost-effective methods to reduce industrial CO_2_ emissions has attracted great attention [1]. High-performance adsorbents with efficient and selective capture of CO_2_ from industrial gas streams are potential candidates [2]. Zeolitic materials with appropriate pore aperture dimensions have an obvious advantage in gas separation based on molecular sieving. For an efficient capture of CO_2_, the zeolite should have high capacity, high selectivity, good hydrothermal stability, and the ease of regeneration [3], which has promoted much research to synthesize new types of zeolite with special structures and decrease the cost of the synthesis process. All-silica Deca-Dodecasil 3 Rhombohedral (Si-DD3R) type zeolite with a two-dimensional pore system shows attractive selectivity in CO_2_ [4]. T-type zeolite was also utilized in the separation of CO_2_/CH_4_ and CO_2_/N_2_ mixtures [5]. In order to overcome the intrinsic limitations of conventional zeolites in CO_2_ capture, recent investigations mostly focused on searching for zeolites with a higher affinity with CO_2_.

Aluminosilicate zeolites of SSZ-13 with the chabazite (CHA) structure were first synthesized by Zones through the hydrothermal method. The SSZ-13 material contains four, six, and eight-membered ring (MR) units to form a three-dimensional pore structure with a large 7.3 Å cage and 3.8 Å 8-MR windows [6]. Due to its unique pore system, SSZ-13 has excellent low- and high-temperature hydrothermal stability, and the production selectivity can be widely used in the methanol-to-olefin (MTO) process [7], catalytic reduction of NOx, NH_3_-selective catalytic reduction (SCR) in the diesel industry, and CO_2_ treatment [8,9]. It was reported that the synthesis of SSZ-13 needs six days, which limited its industrial application. Efforts have been made to decrease the synthesis time, simplify the synthesis process, and improve the activity of the crystals [10]. Improvement of synthesis temperature was demonstrated to be an efficient method to decrease SSZ-13 synthesis time and fast heating prompted crystallization [11]. Adding F^−^ was also used to reduce the synthesis time of SSZ-13; however, it is harmful to the environment [12]. Hollow materials have attracted great attention owing to their unique structure and great potential in adsorption, energy storage, catalysis, and so on [13,14]. Hollow zeolites are particularly outstanding due to their high controlled porosity, thermal stability, and unique shape selectivity, which is significant for gas separation [13,14]. A layer-by-layer self-assembly technique and a controlled dissolution method are often used to prepare hollow zeolite. However, most of the methods are tedious and the alkaline treatment lowers the crystallinity of the final product, which is detrimental to the thermal stability of the formed material [15]. Ultrasonic sonochemistry has been intensively studied and successfully applied to the preparation of materials with unusual properties in recent years. It was supposed that ultrasonics induces the formation, growth, and instantaneously implosive collapse of bubbles in the aqueous solution, which is ready to form local hot spots, decrease crystallization time, and obtain materials with an increased surface area or a hollow structure [16,17]. Hierarchical hollow CuO submicrospheres have been fabricated on a large scale by a facile sonochemical process in the absence of surfactants and additives [18].

Herein, the ultrasonic oscillation was utilized as a direct and facile synthetic route to form hollow zeolite with high crystallinity and adjust the structure of SSZ-13 without adding F^−^. The synthesis time was fixed at 96 h at 433 K for the crystal growth. The effects of seed concentration and ultrasonic time on the structure of SSZ-13 were studied in detail, and CO_2_ adsorption performance was also investigated.

## 2. Experimental

### 2.1. Synthesis of SSZ-13

*N,N,N*-trimethyl-1-adamantanammonium hydroxide (TMAdaOH, 25 wt.%, Alladin, Shanghai, China) was first mixed with NaOH (Kemiou, Tianjin, China) in a beaker with deionized water for 10 min. Then Al(OH)_3_ (Damao, Tianjin, China) was added into the solution with strong stirring. Then colloidal silica (40 wt.%, Sigama-Aldrich, Burlington, MA, USA) was added in slowly. After that, the seed powder was added in the solution with strong stirring. For example, SH-2, the seed powder added, was 0.25 wt.% of the total solution weight. The resulting mixture was treated with ultrasonic oscillation at 40 kHz and 320 W. Then the solution was stirred at room temperature for 6 h to obtain a homogeneous synthesis solution. The solution was transferred to a Teflon reaction kettle for the reaction at 433 K for 96 h. The seed used in this experiment was prepared with conventional method. The Al source was Al(OH)_3_, and the synthesis mixture molar ratio was 20 SDA:20 NaOH:5 Al:100 Si:4400 H_2_O. The preparation procedure of seed was the same as SH-1 except the ultrasonic step. The synthesis conditions are listed in Table 1.

### 2.2. Characterization

X-ray powder diffraction (XRD, Shimadzu XRD-7000S, Shimadzu, Kyushu, Japan) was measured to determine the crystal structure using Cu Kα radiation (40 kV, 40 mA, λ=1.5418 Å). The morphology of the particles was analyzed via scanning electron microscopy (SEM, Nova Nano SEM 450, FEI, Hillsboro, OR, USA) after the samples were coated with gold by sputtering. Transmission electron microscopy (TEM) was analyzed on a Tecnai F30 instrument (FEI, Hillsboro, OR, USA) at 300 kV. The CO_2_ adsorption performance was tested by a specific surface area aperture analyzer (V-Sorb-2800P, APP application, Beijing, China) at 298 K and dried at 823 K for 6 h before testing. The calcined samples with different seed contents were tested with the specific surface area aperture to investigate their structural properties and porous parameters via the N_2_ adsorption/desorption test at 77 K.

## 3. Results and Discussion

### 3.1. Effect of the Amount of Seed on the Sample Structure

**Figure 1:**
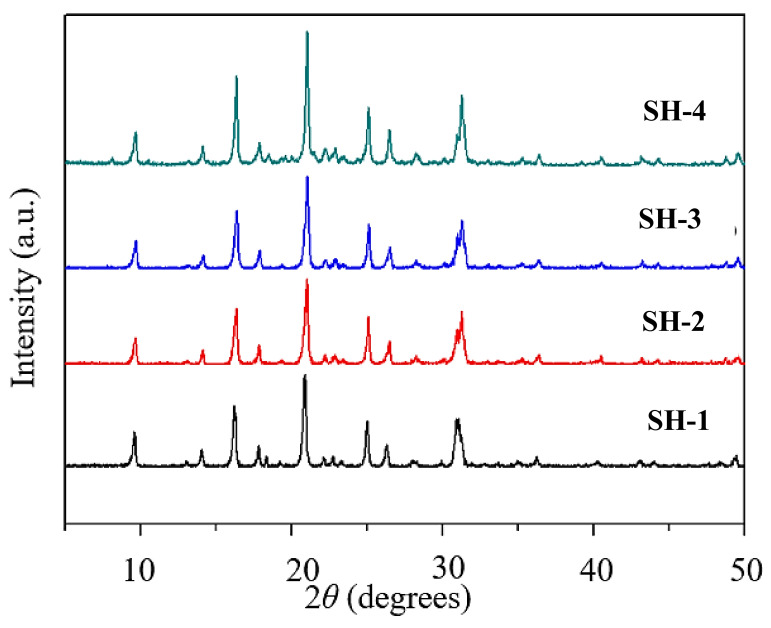
XRD spectra of samples with different seed contents (SH-1, 0 wt.%; SH-2, 0.25 wt.%; SH-3, 0.5 wt.%; SH-4, 1 wt.%).

Figure 1 shows the XRD patterns of samples with different seed contents (from SH-1 to SH-4) prepared by using various amounts of seed. All samples possessed typical diffraction peaks corresponding to the CHA zeolite structure; the strongest diffractions were at 2θ = 9.6°, 16.3°, 21°, 24.9°, and 26.2°. With the increase of seed content, the CHA characteristic peaks changed little while the intensity was improved gradually. This implies that the addition of seed could promote crystallization.

**Figure 2:**
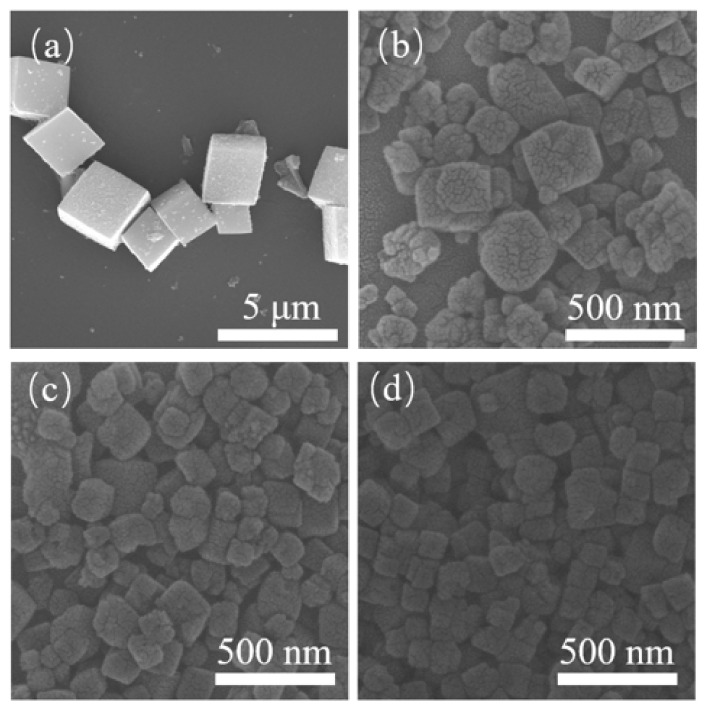
SEM diagrams of samples with different seed contents (**a**, SH-1; **b**, SH-2; **c**, SH-3; **d**, SH-4).

Figure 2 shows the SEM images of the crystalline products. Without seed addition, the crystals showed a typical CHA-type cubic structure. When 0.25 wt.% seed was added, the crystal size decreased below 250 nm. With the further increase of seed content, the crystal size did not change obviously, but particles became uniform. When the seed content was increased to 1 wt.%, the majority of crystals in the sample SH-4 exhibited a cubic morphology and they had a size range of 50–100 nm (Figure 2d). The introduction of seed was beneficial for preparing small particles as shown in Figure 2. It was found that the intensities of the peaks did not only depend on the size of the crystals. With the increase of XRD intensity, the crystal size decreased gradually. Similar results can also be found in references [7,19].

**Figure 3:**
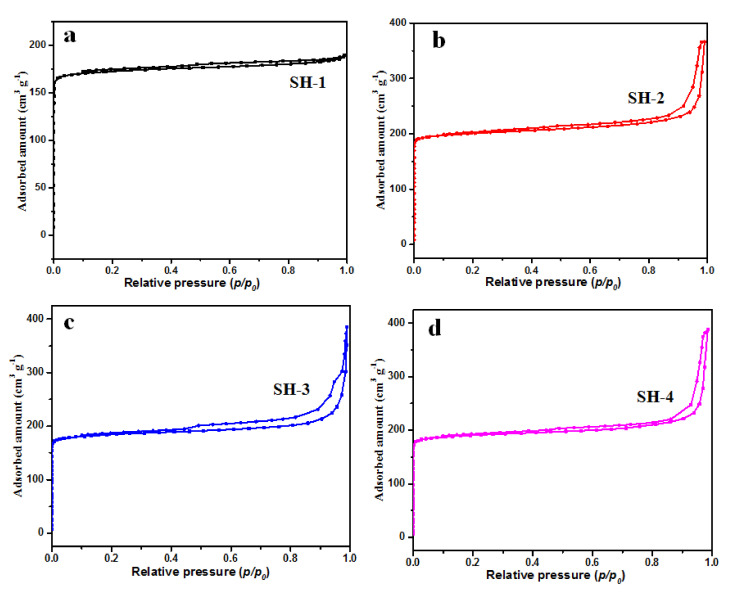
N_2_ adsorption isotherms of (**a**) SH-1, (**b**) SH-2, (**c**) SH-3, and (**d**) SH-4.

**Figure 4:**
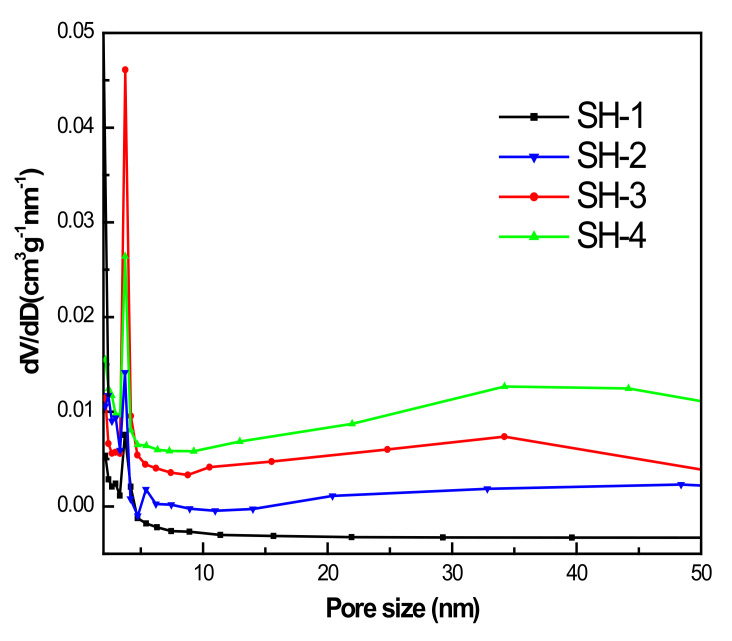
Pore size distribution of samples with different seed contents.

**Figure 5:**
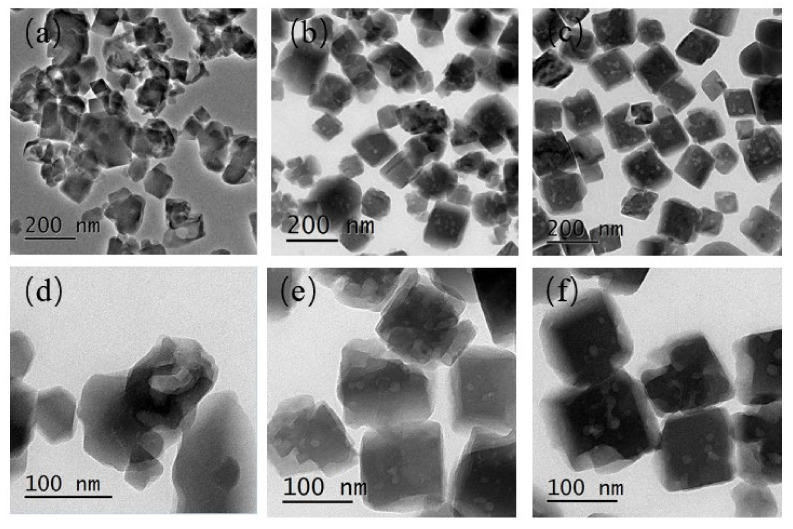
TEM diagrams of SH-2 (**a**,**d**), SH-3 (**b**,**e**), and SH-4 (**c**,**f**).

The calcined samples with different seed contents were selected in order to perform a deeper investigation of their structural properties and porous parameters via the N_2_ adsorption/desorption test at 77 K. Figure 3 presents their N_2_ adsorption isotherms after calcination at 823 K for 6 h. These curves show a steep slope at the relative pressure of 10^−6^< P/P_0_< 0.01, which indicates the presence of micropores, and a hysteresis loop in the 0.40–1.0 relative pressure range relates to the mesoporosities. As shown in the BJH pore size distribution curves in Figure 4, the samples exhibited mesopores with a pore diameter of about 4 nm. The SH-3 sample presented a larger distribution of mesopores at 4 nm. The samples SH-2, SH-3, and SH-4 were also tested with TEM, and the images are shown in Figure 5. All the particles exhibited cubic crystals.

The textural parameters of these structures are listed in Table 2. Without seed added, the sample SH-1 showed very low BET surface area and total pore volume (0.29 cm^3^/g), which is detrimental to efficient gas separation. The SH-2 sample had a larger BET surface area (671.67 m^2^·g^−1^) when compared to SH-3 and SH-4 (610.43 m^2^·g^−1^ and 622.96 m^2^·g^−1^, respectively). However, it showed relative low total pore volume and average pore width. The SH-3 sample showed a high ratio of mesoporous content (56.7%) with suitable total pore volume. In order to prepare hollow SSZ-13 zeolite, 0.5 wt.% seed content was chosen for further research. 

### 3.2. Effect of the Ultrasonic Time on the Sample Structure

**Figure 6:**
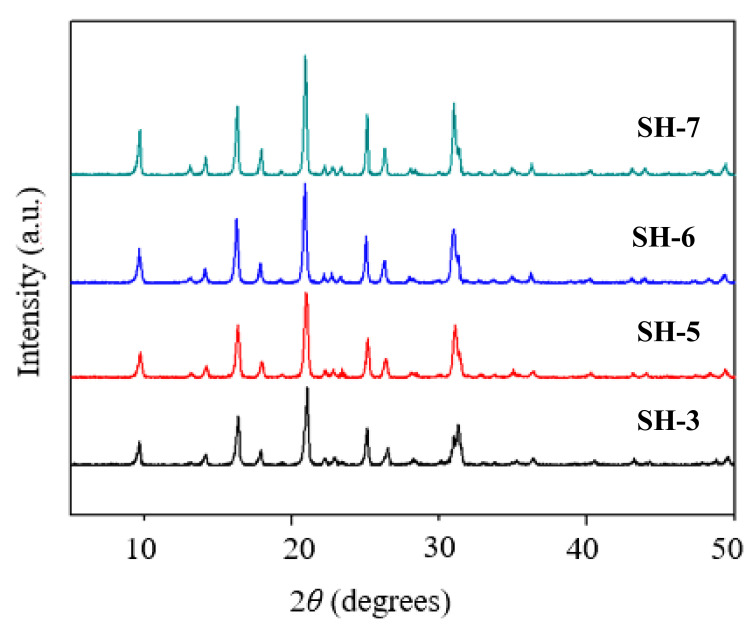
XRD spectra of samples prepared with different ultrasonic times (SH-3, 15 min; SH-5, 30 min; SH-6, 45 min; SH-7, 60 min).

**Figure 7:**
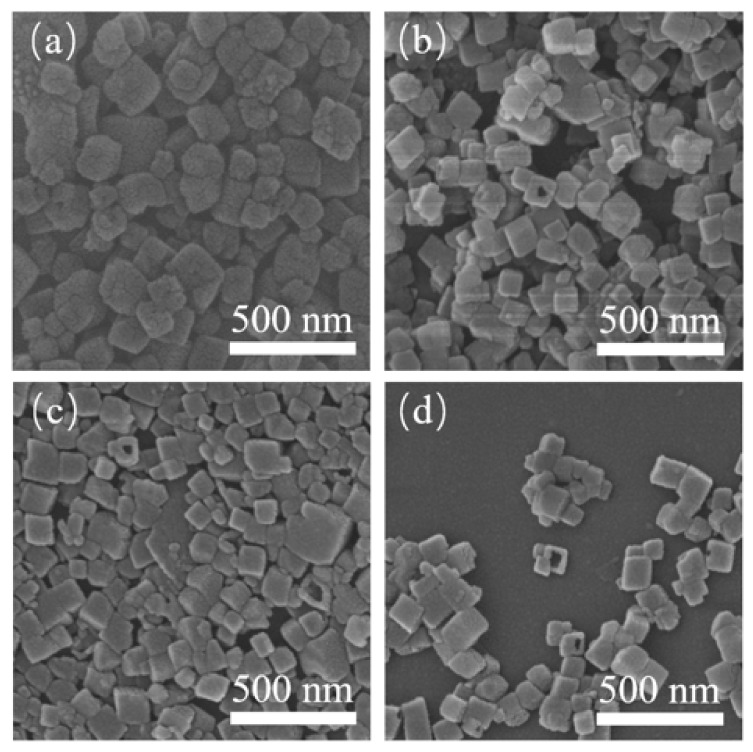
SEM diagrams of SH-3(**a**), SH-5(**b**), SH-6(**c**), and SH-7(**d**).

It has been demonstrated that ultrasound irradiation can promote chemical transformations, improve yields and purity of the products, and shorten the reaction time at mild condition [20]. In this work, seed addition was fixed to 0.5 wt.% and different ultrasonic times on the structure of SSZ-13 were investigated. As shown in Figure 6, all the samples exhibit typical CHA characteristic peaks. When the ultrasonic time was increased from 15 min to 60 min, the intensities of the characteristic peak increased gradually, which implies increased crystallinity. The SEM images of samples prepared with different ultrasonic times are shown in Figure 7. It can be seen that with the increase of ultrasonic time, the particles exhibited typical cubic crystals with decreased particle size. The particle size was only 50 nm when the ultrasonic time was 60 min. Ultrasound with high-energy shock waves was positive to the nucleation rate. Tiny bubbles in the solution grew up and collapsed quickly, which caused super-saturation and promoted homogenization of the solution. Thus, the formed zeolite particles were smaller and more uniform [21,22,23]. As shown Figure 7, the samples showed a hollow structure, and the quantity increased along with the ultrasonic time.

**Figure 8:**
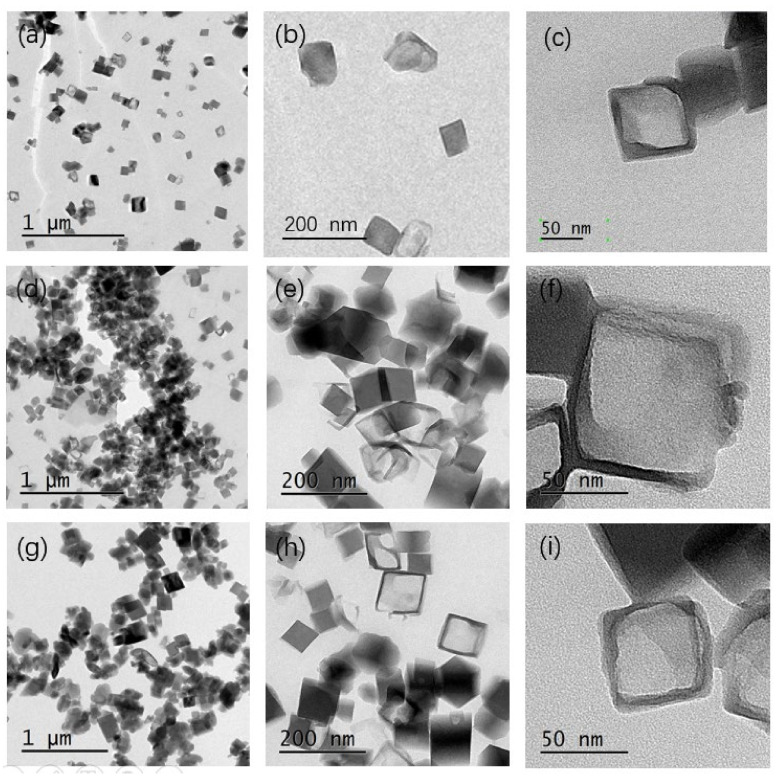
TEM images of SH-5 (**a**–**c**), SH-6 (**d**–**f**), and SH-7 (**g**–**i**).

The samples were also tested with TEM, and the images are shown in Figure 8. All the particles exhibited cubic crystals with a hollow structure. The hollow structure is shown clearly in the TEM images with higher resolution. The hollow SSZ-13 crystals showed a wall thickness of about 5 nm when the ultrasonic time was increased to 60 min as shown in Figure 8i. The collapse of the crystals seen in Figure 8e,h was in accordance with the SEM images.

**Figure 9:**
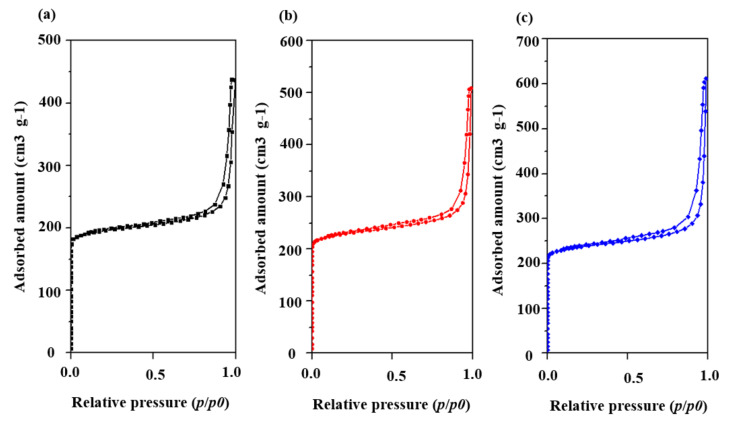
N_2_ adsorption isotherms of (**a**) SH-5, (**b**) SH-6, and (**c**) SH-7.

**Figure 10:**
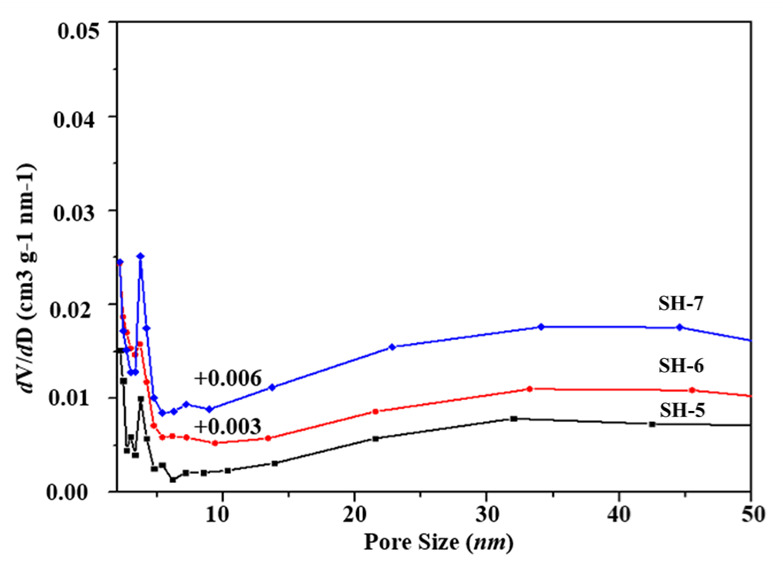
Pore size distribution (SH-5, 30 min; SH-6, 45 min; SH-7, 60 min).

N_2_ adsorption/desorption tests were also carried for the calcined SH-5, SH-6, and SH-7 samples to investigate their structural properties and porous parameters. Figure 9 shows their N_2_ adsorption isotherms after calcination at 823 K for 6 h. These curves with a steep slope at the relative pressure of 10^−6^< P/P_0_ < 0.01 indicated the microporosities in the crystals. The hysteresis loop with the type of H1 at the 0.70–1.0 relative pressure range indicated the presence of mesoporosities. Thus, all the samples possessed microporosities and mesoporosities, which was positive to the adsorption performance. As shown in the BJH pore size distribution curves in Figure 10, all of the samples exhibitedmesoporesat a pore diameter of about 4 nm. The textural parameters of these structures are listed in Table 3. The SH-6 and SH-7 showed very high specific area (763.36 m^2^·g^−1^ and 791.50 m^2^·g^−1^, respectively) with the introduction of ultrasonic treatment. Additionally, the crystals showed high total pore volume, especially the SH-7, which showed 0.95 cm^3^/g of total pore volume and 66.3% content of mesoporosity. Combined with the images of TEM, the crystals with a hollow structure were formed successfully with ultrasonic assistance.

### 3.3. CO_2_ Adsorption Performance of Hollow SSZ-13

**Figure 11:**
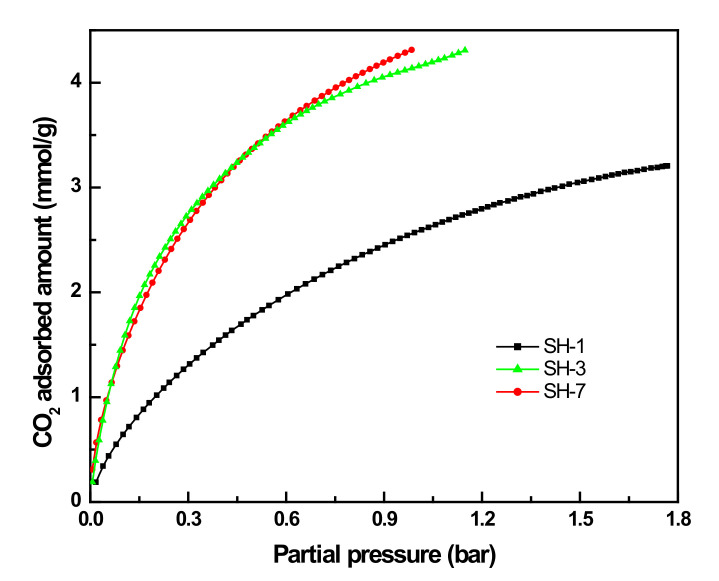
CO_2_ adsorption isotherms of SH-1, SH-3, and SH-7.

SSZ-13 with chabazite topology is a highly stable small-pore zeolite with high CO_2_ capacity, which is an ideal adsorbent for CO_2_ capture. It has high hydrothermal stability, which is easy to regenerate. Figure 11 illuminates the adsorption isotherms for CO_2_ measured at 298 K for SSZ-13 zeolites. As shown in Figure 11, sample SH-1 with low pore volume showed relative low adsorption of CO_2_ compared with samples SH-3 and SH-7. The adsorption of CO_2_ on samples SH-3 and SH-7 increased dramatically starting from very low-pressure (0–0.3 bar) because there were many nanopores in the zeolites. The SH-3 and SH-7 samples with large pore volume and mesoporosity showed higher adsorption performance compared with SH-1 that was prepared without seed addition. At 0.27 bar, the CO_2_-adsorbed amount of SH-7 reached 2.53 mmol/g, which is higher than that of conventional SSZ-13 (1.83 mmol/g). At 1 bar, the sample SH-7 showed a CO_2_ adsorbed amount of 4.24 mmol/g, which is also higher compared with conventional SSZ-13 (3 mmol/g) [2]. Increase of ultrasonic time did not improve the adsorption performance, as shown in Figure 11. The micropores in the zeolites may play a more important role in the CO_2_ capture because CO_2_ may dissolve in the pores and induce higher adsorption amount [24]. The nanosized hollow SSZ-13 showed excellent CO_2_ adsorption performance, which is a potential material for selective capture of CO_2_ from industrial gas streams.

## 4. Conclusions

In conclusion, when studying the effect of seed content and ultrasonic time on the structure of SSZ-13 crystal as well as characterizing the samples with XRD, SEM, N_2_ physical adsorption, and CO_2_ adsorption, it was found that the crystallinity of samples increased with the increase of seed content while the crystal size decreased with the low sized distribution. According to the N_2_ adsorption and TEM characterization, the samples showed a hollow structure with a diameter of 5–20 nm. When the seed content was 0.5 wt.% and ultrasonic time was 60 min, the sample showed CHA-type crystals of about 50 nm with high mesoporosity ratio (66.3%) and a high specific area (791.50 g/m^2^). Compared with SSZ-13 prepared with the conventional method, the SSZ-13 prepared with ultrasonic assistance showed higher CO_2_ adsorption ability. Especially at 298 K and 0.27 bar, the CO_2_-adsorbed amount reached 2.53 mmol/g, which is higher than that of conventional SSZ-13 prepared with conventional methods.

## Figures and Tables

**Table 1:** Synthesis conditions of SSZ-13 molecular sieve.

Sample	Solution Composition SDA:NaOH:Al:Si:H_2_O (wt.% Seed)	Al Source	T/°C	t/h	Ultrasonic Time/Min
SH-1	20:20:5:100:4400 (0)	Al (OH)_3_	160	96	15
SH-2	20:20:5:100:4400 (0.25)	Al (OH)_3_	160	96	15
SH-3	20:20:5:100:4400:0 (0.5)	Al (OH)_3_	160	96	15
SH-4	20:20:5:100:4400:0 (1)	Al (OH)_3_	160	96	15
SH-5	20:20:5:100:4400:0 (0.5)	Al (OH)_3_	160	96	30
SH-6	20:20:5:100:4400:0 (0.5)	Al (OH)_3_	160	96	45
SH-7	20:20:5:100:4400:0 (0.5)	Al (OH)_3_	160	96	60

**Table 2:** N_2_ physical adsorption data of samples with different seed contents.

Sample	S_BET_ (m^2^/g)	Total Pore Volume ^1,3^ (cm^3^/g)	Mesoporous Volume ^2^ (cm^3^/g)	Micropore Volume ^1^ (cm^3^/g)	Average Pore Width (nm)	Content of Mesoporous (%)
SH-1	574.91	0.29	0.04	0.25	6.393	13.8
SH-2	671.67	0.57	0.28	0.28	16.74	49.1
SH-3	610.43	0.60	0.34	0.28	18.50	56.7
SH-4	622.96	0.60	0.32	0.29	22.44	53.3

^1^ Calculated with t-plot method. ^2^ Calculated with BJH method. ^3^ P/P_0_ = 0.9918, 0.9885, 0.9906, 0.9947.

**Table 3:** N_2_- physical adsorption data of SH-5 to SH-7.

Sample	S_BET_ (m^2^/g)	Total Pore Volume ^1,3^ (cm^3^/g)	Mesoporous Volume ^2^ (cm^3^/g)	Micropore Volume ^1^ (cm^3^/g)	Average Pore Width (nm)	Content of Mesoporous (%)
SH-5	653.45	0.68	0.41	0.26	21.53	60.3
SH-6	763.36	0.78	0.47	0.30	19.11	60.3
SH-7	791.50	0.95	0.63	0.31	21.96	66.3

^1^ Calculated with t-plot method. ^2^ Calculated with BJH method. ^3^ P/P_0_ = 0.9977, 0.9907, 0.9889.
